# Aging Failure Mechanism of Transformer Bushing Sealing Rings Under Multi-Factor Effect

**DOI:** 10.3390/ma19030614

**Published:** 2026-02-05

**Authors:** Wei Liang, Huijie Li, Zengchao Wang, Yuan La, Yao Yuan, Fanghui Yin, Liming Wang

**Affiliations:** 1 Shenzhen International Graduate School, Tsinghua University, Shenzhen 518055, China; liangw23@mails.tsinghua.edu.cn (W.L.); lihj24@mails.tsinghua.edu.cn (H.L.); wanglm@sz.tsinghua.edu.cn (L.W.); 2 China Southern Power Grid Co., Ltd., Guangzhou 510623, China; wangzc1@csg.cn (Z.W.); ylacsg@163.com (Y.L.); 3 Electric Power Research Institute of China Southern Power Grid, Guangzhou 520080, China

**Keywords:** transformer bushing, sealing ring, multi-factor aging, failure mechanism, nitrile rubber, fluoroelastomer

## Abstract

The aging and failure of transformer bushing seals under multi-factor effects are significant causes of oil leakage incidents. However, their failure mechanisms under combined environmental stressors remain inadequately understood. This study presents a comprehensive investigation into the aging behavior and failure mechanisms of nitrile rubber (NBR) and fluoroelastomer (FKM) sealing materials subjected to single and multi-factor aging conditions, including thermo-oxidative, hygrothermal, hygrothermal–compression, and hygrothermal–compression–salt environments. NBR undergoes severe degradation under multi-factors, dominated by additive loss and molecular chain crosslinking. At high temperatures, large-scale molecular chain scission occurs, along with increased compression set, microscopic morphological damage, and filler precipitation. In contrast, FKM exhibits excellent stability thanks to its C-F main chain. Stress synergy significantly accelerates the failure of both materials. These findings highlight the need for multivariate analysis to support reliable condition assessment and lifetime prediction and to inform sealing material selection and proactive grid maintenance.

## 1. Introduction

Power transformers are cornerstone components of electrical power systems, ensuring the reliable and efficient transmission and distribution of electricity [1]. The integrity of these massive assets is critical to grid stability; their failure can cause significant economic impacts and prolonged power outages. Within the intricate architecture of a transformer, the high-voltage bushings play a vital role, providing essential electrical insulation and mechanical support while allowing current-carrying conductors to pass through the grounded transformer tank [2]. Despite ongoing advancements in transformer technology and diagnostic monitoring, bushing failures remain a primary cause of transformer outages [3,4]. A frequently under-researched yet critical factor contributing to this vulnerability is the long-term degradation of the sealing systems used within these bushings [5].

The operational life of a transformer bushing fundamentally relies on the ability of its sealing system to maintain a hermetic barrier [6,7,8]. These seals, typically composed of polymeric materials like nitrile rubber (NBR), silicone rubber (SIR), Fluororubber (FKM), or ethylene propylene diene monomer (EPDM), prevent the detrimental ingress of moisture and ambient air into the internal oil-paper insulation system, while simultaneously preventing the escape of insulating fluid. Over decades of service, these polymeric gaskets are exposed to a demanding combination of environmental and operational stresses that inevitably lead to physical and chemical degradation, compromising the seal’s integrity. For some sealing rings in coastal areas of southern China, after aging in the natural environment, the hardness even exceeds 90 HD, and the intensities of some characteristic peaks in Fourier transform infrared (FTIR) spectroscopy analysis decrease by more than 90% [9,10].

The existing literature on transformer reliability and insulation aging has extensively documented the degradation of the oil-paper insulation system, resulting in established life expectancy curves and diagnostic criteria published by major standards organizations such as IEEE and IEC. However, specific research focused on the long-term performance and failure mechanisms of the sealing materials themselves remains sparse. Current condition monitoring techniques for bushings, which often rely on electrical measurements such as capacitance and power factor (tan δ), primarily detect advanced stages of insulation degradation rather than providing early warnings of seal failure.

Polymer science literature provides insight into several key stressors that accelerate material aging in harsh environments, which is directly applicable to transformer bushing seals [6,11,12,13]. Thermal aging is widely recognized as the most significant operational stressor. The internal operating temperatures of bushings, influenced by conductor current loading and ambient conditions (which can exceed the standard 40 °C maximum ambient temperature rating), accelerate the chemical kinetics of polymer degradation. This process, involving chain scission and cross-linking [13,14], leads to a permanent change in mechanical properties such as elasticity and compression set, ultimately resulting in a loss of the crucial contact pressure required for an effective seal.

In concert with thermal stress, oxidative aging (or thermal-oxygen aging) is a critical degradation pathway [15,16,17]. The presence of oxygen, even at low concentrations within the sealed system or external atmosphere, significantly accelerates the aging process at elevated temperatures. This leads to the formation of polar functional groups (such as carbonyls), which are detected via methods like FTIR spectroscopy [14,18,19,20], further compromising the material’s mechanical and chemical stability.

Furthermore, external environmental factors play a substantial role in degrading exposed sections of the seal. Humidity is a particularly insidious threat. Long-term exposure to moisture can lead to the hydrolytic degradation of certain polymers and serve as a primary pathway for contamination once a seal is breached [21,22,23,24,25]. Moisture contamination within the bushing drastically reduces the dielectric strength of the insulating fluid and paper, accelerating their aging and increasing the risk of partial discharges and electrical breakdown. The effects of moisture on the polymer, including plasticization and swelling, can further reduce its sealing effectiveness. In coastal or industrial environments, exposure to salt fog and other atmospheric pollutants can deposit conductive layers on porcelain or composite surfaces.

**Figure 1:**
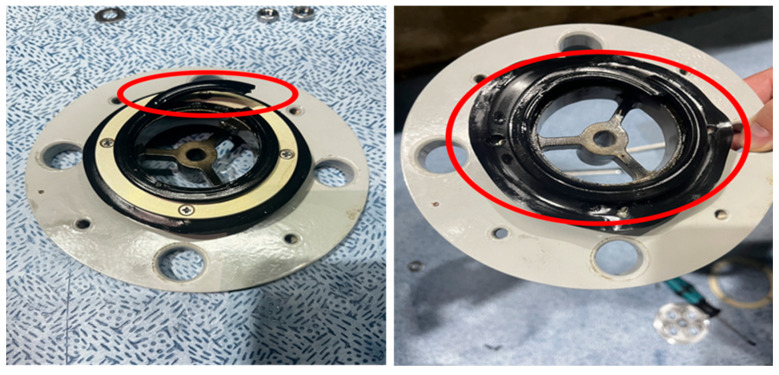
The transformer seal failure.

The synergistic interplay of these multiple stressors—humid, salt, high temperature, and thermal-oxygen aging—creates a complex degradation landscape that cannot be fully understood through single-factor testing alone. The current lack of a detailed understanding of these combined aging mechanisms, along with the absence of specific, reliable diagnostic criteria for real-time monitoring of seal health, poses a significant risk to the reliability of aging transformer fleets. A transformer seal failure is illustrated in Figure 1. The red parts indicate the damaged seal ring.

This article aims to bridge this knowledge gap by providing a comprehensive investigation into the multi-stress aging performance of common polymeric sealing materials used in high-voltage transformer bushings. Using a combination of accelerated laboratory aging tests under controlled conditions (thermal, oxidative, humid, and salt fog) and advanced material characterization techniques, this study seeks to elucidate the primary degradation mechanisms. The research will provide valuable insights for condition assessment and asset management strategies, enabling utility operators to move from reactive maintenance to proactive management of bushing sealing systems, thus enhancing overall grid reliability and safety.

## 2. Experimental Setup

### 2.1. Sample Preparation

**Figure 2:**
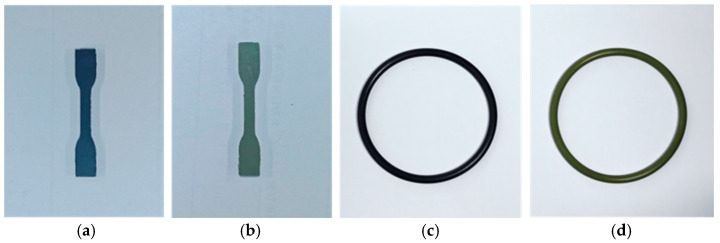
The transformer seal samples. (**a**) NBR dumbbell-shaped specimen, (**b**) FKM dumbbell-shaped specimen, (**c**) NBR sealing ring, (**d**) FKM sealing ring.

The nitrile rubber (NBR) and fluoroelastomer (FKM) sealing materials used in this study were procured from Jiangsu Shenma Electric Power Co., Ltd., Nantong, China. Dumbbell-shaped specimens were prepared in accordance with GB/T 528-2009 using a JCP-25 die-cutting machine from Yangzhou Jingyi Testing Machinery Co., Ltd., Yangzhou, China [26]. The specimens have a gauge length of 10 mm, a thickness of 2 mm, and an overall length of 50 mm. Additionally, O-rings with an inner diameter of 90 mm, an outer diameter of 102 mm, and a cross-sectional diameter of 6 mm were employed. Appearances of the NBR and FKM samples are presented in Figure 2.

### 2.2. Experimental Arrangement

**Figure 3:**
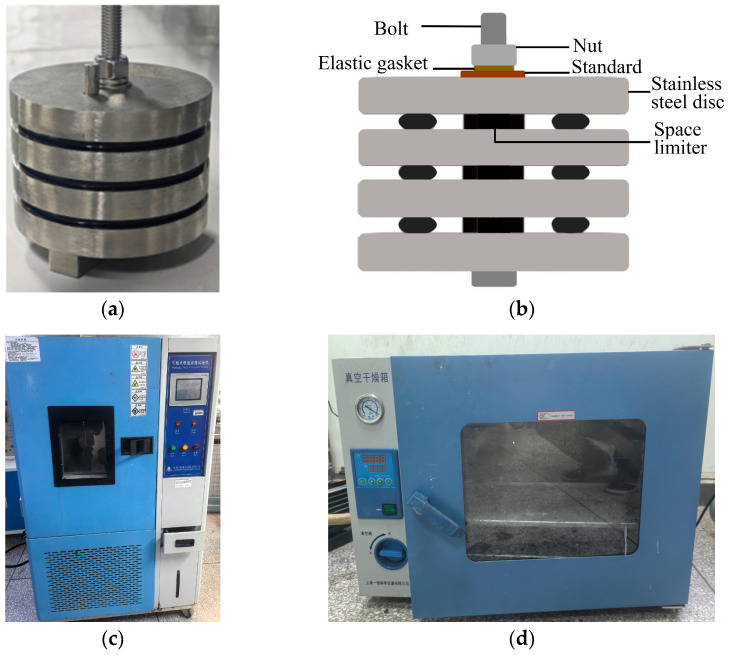
The experimental setup. (**a**) External View of the multi-layer compression structure, (**b**) Cross-sectional view of the multi-layer compression structure, (**c**) The constant temperature and humidity chamber, (**d**) The thermal-oxygen aging chamber.

The experimental setup is illustrated in Figure 3. The independently designed multi-layer compression structure, as depicted in the Figure 3a assembly diagram, incorporates multiple components including limiters of varying heights (4.2 mm, 4.5 mm, 4.8 mm) to simulate compression rates of 20%, 25%, and 30%, alongside locating pins, metal discs, bolts, nuts, and spring washers. This configuration enables simultaneous testing of multiple sealing rings, thereby enhancing testing efficiency.

The experimental setup primarily comprises a constant-temperature, constant-humidity chamber and a thermal-oxygen aging chamber.

### 2.3. Experimental Method

Based on relevant studies [6,7,8,9,10] and the previous analysis of regularity in single-factor research, the determination of test conditions and the design of the test structure were conducted. A multi-layer compression-sealing structure was designed to conduct dual-factor thermo-oxidative and compression-aging tests on nitrile and fluorocarbon rubber seals. These tests were performed at three temperatures (90 °C, 105 °C, 120 °C) and three compression ratios (20%, 25%, 30%), as well as non-compression thermal-oxygen aging tests at 120 °C. The aging duration was 80 days. The aging chamber complied with GB/T 3512-2014 standards [27]. Seals were retrieved for performance testing at various aging intervals (0 days, 10 days, 20 days, 40 days, 80 days); longer aging times allow for better measurement of changes in aging performance.

To investigate the influence of humidity and compression factors on aging failure mechanisms, dual-factor humidity-heat ageing tests were conducted on dumbbell-shaped specimens and rubber seals made from nitrile and fluorocarbon rubber. A multi-layer compression structure was employed for the seals to introduce the compression factor. Tests were conducted at 80 °C, 85% relative humidity, and compression rates of 20%, 25%, and 30% using an RHP-64AT programmable constant temperature and humidity chamber from Hireale Testing Equipment Co., Ltd., Dongguan, China to simulate the humid heat environment. The total aging duration was 60 days, with material samples retrieved at 0 d, 10 d, 20 d, 40 d, and 60 d for corresponding testing.

To simulate the combined effects of salt exposure, dumbbell-shaped specimens with nitrile rubber and fluorocarbon rubber sealing rings were subjected to combined humidity, heat, and salt aging. A 5% NaCl saline solution was uniformly sprayed daily using a sprayer to simulate salt effects.

### 2.4. Property Measurement

(1) Mass Loss Measurement

The mass of the samples was recorded before aging (denoted as $m_{0}$). To quantify the degradation extent, the mass change rate (η) was calculated after each aging interval. The mass measured after different aging periods is denoted as *m_i_*. Three groups of parallel samples were used, and the average value was calculated. The mass change was systematically recorded and analyzed at various stages of the aging process.

(1) $$\eta =\frac{m_{i}-m_{0}}{m_{0}}\times 100\%$$

(2) Compression Set Measurement

The compression set (CS), which indicates the permanent deformation of an elastomer after compression, was calculated using the following equation [28]:

(2) $$\mathrm{CS}=\frac{h_{0}-h_{1}}{h_{0}-h_{s}}\times 100\%$$

where *CS* is the compression set (%), *h*_0_ is the initial thickness (mm) of the specimen, *h*_1_ is the final thickness (mm) after recovery, and *h_s_* is the height (mm) of the compression spacer. A *CS* value of 100% indicates that the seal completely failed to recover from the compressive strain, while 0% signifies full recovery. Values exceeding 100% can be obtained if the sample shrinks during aging.

(3) Surface morphology analysis

The surface integrity and overall condition of the seals were first examined macroscopically using a digital macro camera. For detailed microstructural analysis, a HITACHI SU8010 cold-field emission scanning electron microscope (SEM) from Hitachi, Ltd., Tokyo, Japan was employed. SEM observations were conducted on different sections of the seals to characterize the morphological changes induced by varying aging durations and compression ratios. Information is obtained through the acquisition of secondary electrons (SE) and backscattered electrons (BSE). To enhance the electrical conductivity of rubber materials, tests were conducted after gold sputtering.

(4) Energy Dispersive X-ray Spectroscopy (EDS) analysis

Elemental analysis was performed using an IXRF Model 550i Energy Dispersive Spectrometer (EDS) from IXRF Systems, Inc., Round Rock, TX, USA attached to the SEM system. This combined SEM-EDS approach was used to determine the relative elemental composition of the samples at the microstructural level.

(5) Thermal Analysis

Simultaneous Thermogravimetric Analysis (TGA) and Differential Scanning Calorimetry (DSC) were performed using a NETZSCH STA 449 F3 instrument from Hitachi, Ltd., Tokyo, Japan. The measurements were conducted to investigate the thermal stability of the seals under various aging times, compression ratios, and temperatures. Experiments were conducted in either a nitrogen or an oxygen atmosphere [19] at a gas flow rate of 50 mL/min. The temperature was ramped from room temperature to 800 °C at a constant heating rate of 20 °C/min. This technique enabled monitoring of mass loss (TGA), the rate of mass loss (Derivative Thermogravimetry, DTG), and thermal transitions (DSC) throughout the programmed temperature scan.

(6) Fourier transform infrared spectroscopy

FTIR analysis is a commonly used method for studying the aging and failure process of rubber sealing materials [29,30,31]. Nicolet iS50 Fourier transform infrared (FTIR) spectrometer from NETZSCH-Gerätebau GmbH, Selb, Germany was employed, with tests conducted in attenuated total reflection (ATR) mode. The scanning range was 4000–500 cm^−1^, resolution was 4 cm^−1^, and the number of scans was 32. Special attention was paid to changes in the intensities of characteristic carbonyl, C-F, and C=C peaks to characterize the material’s oxidation state and chemical bond cleavage.

## 3. Results and Discussion

### 3.1. Mass Loss Evolution Under Different Aging Conditions

#### 3.1.1. Mass Loss Evolution Under Thermo-Oxidative Aging

**Figure 4:**
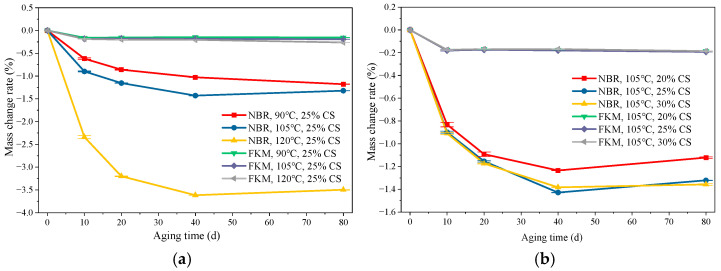
The mass change results. (**a**) Mass change at different temperatures (**b**) Mass change at different compression ratios.

The mass change rate of the seals as a function of aging time under thermo-oxidative-compressive dual-factor aging is shown in Figure 4. Because both thermal oxidation and compression generally lead to mass reduction, the mass change rate is negative.

NBR exhibited a substantial mass loss during aging, whereas FKM showed minimal change. As shown in Figure 4a, at a constant compression ratio of 25%, the mass of both rubber types decreased rapidly during the initial aging stage, reaching a maximum loss around 40 days before stabilizing. The mass loss of NBR increased significantly with rising temperature, while that of FKM was largely unaffected by temperature variations. The pronounced mass loss in NBR is primarily attributed to the migration and subsequent volatilization of additives, such as plasticizers and antioxidants, from the rubber matrix into the environment during thermo-oxidative aging. The rapid depletion of antioxidants, in particular, accelerates the severe degradation of the rubber. In contrast, the minimal mass change in FKM, likely originating from the volatilization of minor plasticizers, underscores its superior thermal stability.

Figure 4b illustrates the mass change in the seals at a constant temperature of 105 °C. NBR mass loss increased with higher compression ratios. However, this effect became less pronounced once the compression ratio exceeded 25%. This phenomenon can be mainly explained by the sufficient oxygen supply from the seal’s sides, which adequately diffuses to the areas covered by the metal plates. The stress concentration induced by compression likely accelerated the aging process, facilitating greater volatilization of additives such as plasticizers and leading to increased mass loss. Conversely, the mass loss of FKM is essentially insensitive to the compression ratio, indicating its excellent resistance to compression and thermal oxidative aging.

#### 3.1.2. Mass Loss Evolution Under Hygrothermal–Salt–Compression Aging

**Figure 5:**
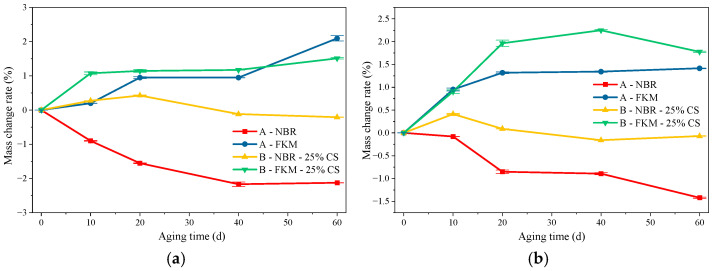
The Mass change results under hygrothermal–salt–compression aging. (**a**) Mass change under hygrothermal–compressive aging (**b**) Mass change under hygrothermal–salt–compression aging.

Figure 5 illustrates the specimen configurations, where ‘A’ denotes the dumbbell-shaped specimen and ‘B’ represents the O-ring within the multi-layer compression assembly. As shown in Figure 5a, the mass of NBR decreased under hygrothermal aging alone. However, with the superimposed effect of compression (25% compression ratio), the mass of the NBR seal initially increased, peaked at 20 days, and subsequently decreased, approaching its initial value by 60 days. This trend is attributed to competing mechanisms: the initial mass gain resulted from water absorption via physical diffusion and hydrogen bonding. At the same time, the migration and dissolution of small-molecule additives, such as plasticizers, caused the subsequent loss.

From Figure 5b, it is evident that the introduction of salt ions accelerated this process, shifting the mass peak forward by 10 days, after which a rapid decline ensued. The adsorbed salt particles increased the osmotic pressure, thereby drawing more water into the polymer and exacerbating swelling. Concurrently, the high ion permeability intensified interfacial reactions, leading to severe surface erosion and, consequently, accelerating mass loss. In contrast, the mass of FKM increased significantly after both hygrothermal and salt spray humid heat aging, indicating a limited influence of moisture and salt. The mass gain is primarily attributed to moisture adsorption, salt deposition, and corrosion products formed on the metal plates. The superior stability of FKM stems from its backbone, which consists predominantly of high-bond-energy C-F bonds and contains minimal unsaturated sites, thereby conferring far greater resistance to degradation than NBR. This inherent stability prevents significant mass loss from chain scission in hygrothermal environments. While the presence of salt enhanced moisture absorption and led to some deposition, a slight mass reduction was observed in later stages due to the leaching of minor additives (e.g., curing agents and plasticizers). Nevertheless, the final mass remained significantly higher than the initial value.

### 3.2. Compression Set Under Different Aging Conditions

#### 3.2.1. Compression Set Evolution Under Thermo-Oxidative Aging

**Figure 6:**
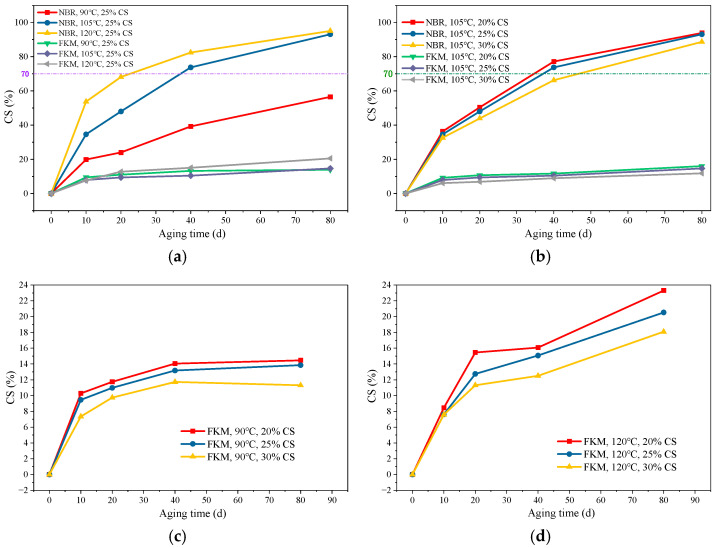
The CS change results under hygrothermal–salt–compression aging. (**a**) CS rate change at different temperatures, (**b**) CS rate change at 105 °C under different compression ratios, (**c**) CS rate change at 90 °C under different compression ratios of FKM, (**d**) CS rate change at 120 °C under different compression ratios of FKM.

Compression set (CS) characterizes the extent to which a seal fails to recover its original shape after the compressive load is released. The CS results for NBR and FKM after thermo-oxidative aging are presented in Figure 6.

The results in Figure 6a reveal that the CS of NBR increases progressively with aging time under thermo-oxidative conditions. This increase is primarily due to the volatilization of plasticizers and to small molecules generated by oxidative chain scission during high-temperature aging. Considering the effect of compressive stress, temperature (90 °C, 105 °C, 120 °C) significantly influences CS at a 25% compression ratio, with elevated temperatures substantially increasing CS. During the early aging stage (≤10 days), the rate of CS increase is also markedly faster at higher temperatures, indicating an accelerated aging process. This suggests that high temperature not only exacerbates the volatilization of plasticizers and oxidative cross-linking of the main chains but also induces more severe chain scission, generating small molecular chains or branches. The low molecular weight products generated during its aging mainly include volatile decomposition products of plasticizers, C4~C8 short-chain hydrocarbons produced by polymer backbone scission, and carboxylic acids formed through oxidation reactions.

As shown in Figure 6b, at the same temperature, the CS of the NBR seal at a 20% compression ratio is consistently higher than that at 25% and 30%. Compared with existing studies, this study more precisely demonstrates the effects of different compression ratios. The reason is that lower compression allows greater mobility of the molecular chains, thereby accelerating the oxidation reaction. This simultaneously intensifies both cross-linking and chain scission, ultimately leading to a denser yet more fragile network structure at high temperature. This results in rubber hardening, loss of elasticity, and, consequently, increased compression set.

For FKM, the CS also gradually increases with aging time, but the change is considerably smaller than that for the NBR seal. As shown in Figure 6c,d, the change in CS is greater at 120 °C than at 105 °C and 90 °C. At the same temperature, the CS values for compression ratios of 20%, 25%, and 30% decrease. FKM exhibits excellent heat resistance. During high-temperature aging, it primarily undergoes oxidation at the chain ends and cross-linking of long main chains, with large-scale oxidation and cross-linking being limited. The main groups undergoing chain-end oxidation are hydrogen-containing groups such as –CF_2_H and –CH_2_–, along with a small amount of unsaturated end groups. Upon oxidation, these groups form polar products like carboxyl groups, a process confined locally to the chain ends due to the high stability of the backbone C–F bonds. Coupled with the volatilization of some additives, such as plasticizers, this results in only a slight decrease in elasticity and a marginal increase in compression set.

#### 3.2.2. Compression Set Evolution Under Hygrothermal–Salt–Compression Aging

To investigate the effects of hygrothermal and salt on the mechanical properties of the seals, tensile tests were conducted on NBR and FKM dumbbell-shaped specimens before and after hygrothermal and hygrothermal–salt aging. The results are summarized in Table 1.

The tensile properties of NBR were analyzed. During hygrothermal aging, the elongation at break (Eb) gradually decreased with aging time. The initial Eb before aging was 235.4%, which decreased to 205.56% after 60 days of hygrothermal aging and further reduced to 185.12% under hygrothermal–salt conditions. The initial tensile strength (TS) was 17.37 MPa. It exhibited a slight increase after hygrothermal aging but a minor decrease following hygrothermal–salt aging.

In contrast, the FKM showed superior stability. Its Eb remained in the range of 260–275% without a distinct downward trend, while the TS fluctuated marginally between 11 MPa and 13 MPa. This indicates minimal degradation, attributable to FKM’s stable backbone structure and excellent anti-aging properties.

The underlying mechanism for these trends is that the flexibility and mobility of molecular chains intrinsically determine Eb. Hygrothermal aging induces oxidative cross-linking, increasing cross-link density, restricting chain segment movement, and consequently reducing Eb. The presence of salt ions accelerates this process. Meanwhile, TS reflects the material’s resistance to tensile failure. Both hygrothermal aging and salt spray testing demonstrate a synergistic effect on the degradation of the mechanical performance of the sealing rings.

**Figure 7:**
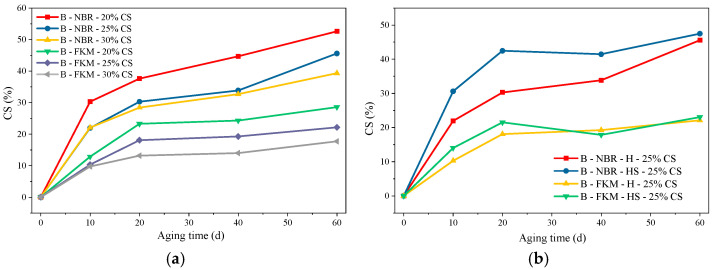
The CS change results under hygrothermal–salt–compression aging. (**a**) CS change under hygrothermal–compression aging with different compressive ratios. (**b**) CS change under different aging factors.

The compression set of the seals after hygrothermal–salt–compression aging is presented in Figure 7.

The results in Figure 7a indicate that the compression set of the nitrile rubber (NBR) seal increased rapidly during the initial aging stage and then plateaued, reaching or approaching 50% after 60 days of aging. Compared with other studies, a longer aging time enables more accurate quantification of the material’s performance changes as it approaches failure. In contrast, the FKM seal exhibited a much smaller change in compression set than NBR, with values ranging from 15% to 30% after the same aging period.

The results in Figure 7b demonstrate that the NBR seal underwent a more pronounced increase in compression set under the combined hygrothermal–salt–compression condition than under hygrothermal–compression aging alone. This accelerated degradation is primarily attributed to the rapid formation of cross-links, which reduces the material’s resilience and elasticity. The leaching of anti-aging agents and plasticizers further exacerbates the aging process. Moreover, the penetration of a salt solution intensifies structural deterioration, facilitating the ingress of additional moisture and salt ions into the rubber matrix.

### 3.3. Surface Morphology Analysis Under Different Aging Conditions

#### 3.3.1. Surface Morphology Analysis Under Thermo-Oxidative Aging

**Figure 8:**
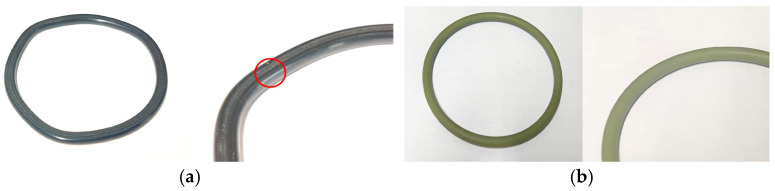
Macro morphology of the sealing ring after thermo-oxidative-compressive aging. (**a**) NBR (**b**) FKM.

Figure 8 shows the morphology of the NBR and FKM seals before and after thermo-oxidative-compressive aging. As can be seen in the red area circled in Figure 8a, the NBR seals suffered severe degradation, becoming hard, brittle, deformed, and covered with white spots—after thermo-oxidative-compression aging. The FKM seals, however, demonstrated superior stability, showing no deformation and only minimal changes in elasticity, confirming their excellent resistance to this aging condition.

**Figure 9:**
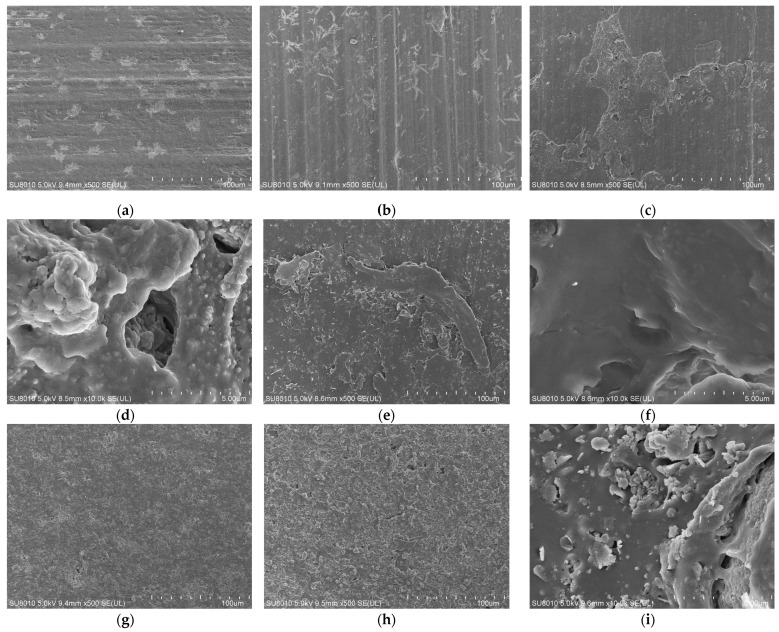
Surface morphology of the sealing ring after thermo-oxidative-compressive aging. (**a**) NBR-NEW. (**b**) NBR-120 °C-20 d. (**c**) NBR-120 °C-80 d ×500. (**d**) NBR-120 °C-80 d ×10,000. (**e**) NBR-120 °C-25%-80 d ×500. (**f**) NBR-120 °C-25%-80 d ×10,000. (**g**) FKM-NEW. (**h**) FKM-120 °C-20%-80 d ×500. (**i**) FKM-120 °C-20%-80 d ×10,000.

Samples were taken from the surface of the seals to observe the micro-morphology of NBR and FKM under varying aging durations, compression ratios, and locations. Observations were primarily conducted at magnifications of ×500 and ×10,000, as shown in Figure 9.

Analysis of the NBR revealed a uniform and smooth surface in the unaged sample. With increasing aging time and temperature, the seal surface developed progressively more micro-pores and flaky peeling, becoming increasingly rough with a greater number of cracks. After aging at 120 °C for 80 days, the surface was severely damaged, characterized by significantly enlarged voids. This deterioration is attributed to the volatilization of antioxidants and plasticizers, coupled with the degradation of the rubber matrix, in which intensified oxidation led to extensive cross-linking and chain scission. In contrast, the FKM, with its relatively stable chemical properties, showed no severe surface damage. However, some matrix disruption and filler exposure were still evident after aging.

#### 3.3.2. Surface Morphology Analysis Under Hygrothermal–Salt–Compression Aging

**Figure 10:**
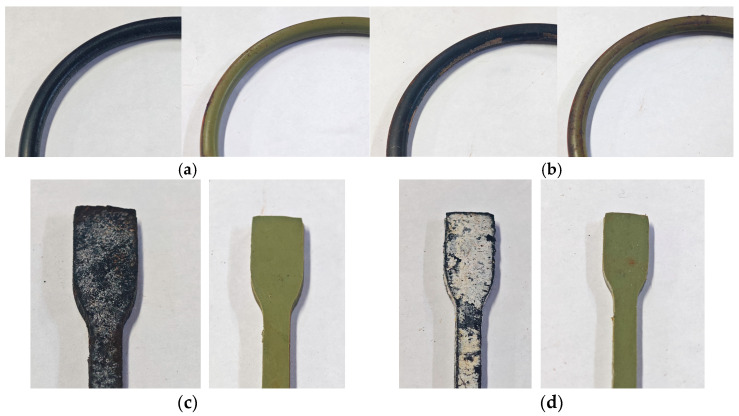
Macro morphology of the sealing ring after hygrothermal–salt–compression aging. (The left sample is NBR, the right sample is FKM.) (**a**) hygrothermal–compression aging. (**b**) hygrothermal–salt–compression aging. (**c**) hygrothermal aging. (**d**) hygrothermal–salt aging.

Figure 10 presents the macroscopic morphology of NBR and FKM specimens (both dumbbell-shaped and O-ring) after aging.

For NBR, surface whitening and fine cracks were observed following hygrothermal and hygrothermal–compression aging. On dumbbell specimens, these features were uniformly distributed, whereas on O-rings they were concentrated on the free surfaces (sides), with significantly fewer marks on the compressed surfaces. This indicates spatially non-uniform degradation due to moisture exposure. The introduction of salt markedly intensified the whitening: dumbbell specimens were almost entirely covered with white powder, while O-rings exhibited greatly expanded whitened areas on the sides, accompanied by rust stains. In simulated sealing structures, whitening even extended to the compressed surfaces, demonstrating the pronounced influence of salt on macroscopic morphology.

In the case of FKM, rust formation occurred after aging, most prominently at the interface between the compressed surface and the free surface, where moisture, air, and the metal disk interacted, leading to rust accumulation. The overall surface became rougher with prolonged aging, developing more irregular indentations.

**Figure 11:**
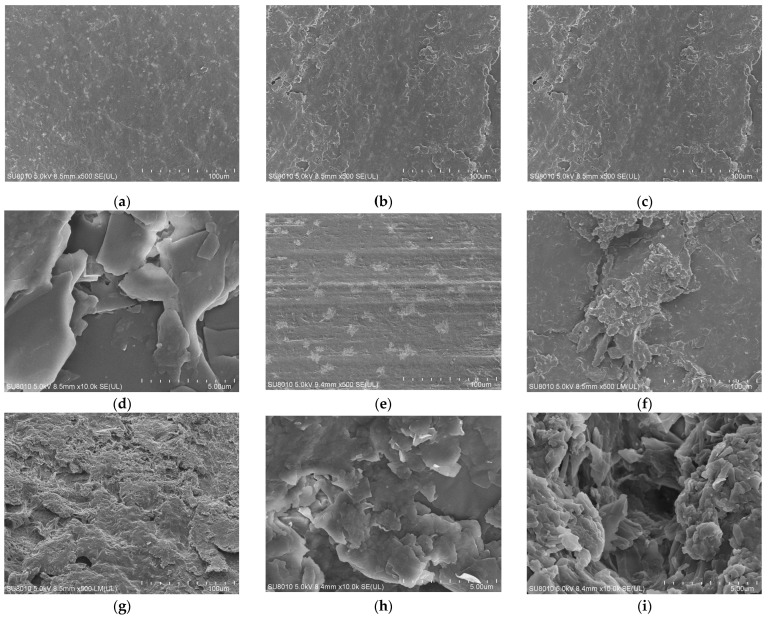
Surface morphology of NBR after hygrothermal–salt–compression aging. (**a**) NBR-A-NEW. **(b**) NBR-A-Hygrothermal-60 d. (**c**) NBR-A-Hygrothermal-Salt-60 d ×500. (**d**) NBR-A-Hygrothermal-Salt-60 d ×10,000. (**e**) NBR-B-NEW. (**f**) NBR-B-Hygrothermal-25%-60 d ×500. (**g**) NBR-B-Hygrothermal-Salt-20%-60 d ×500. (**h**) NBR-B-Hygrothermal-20%-60 d ×10,000. (**i**) NBR-B-Hygrothermal-Salt-20%-60 d ×10,000.

Micro-morphology of NBR dumbbell and seal specimens before and after hygrothermal and hygrothermal–salt aging was examined, with the results shown in Figure 11. The unaged NBR specimens exhibited relatively smooth and flat surfaces. After hygrothermal aging, flaky peeling and cracks appeared. With the addition of salt, the surfaces developed more protrusions and fragments, along with numerous expelled filler particles.

**Figure 12:**
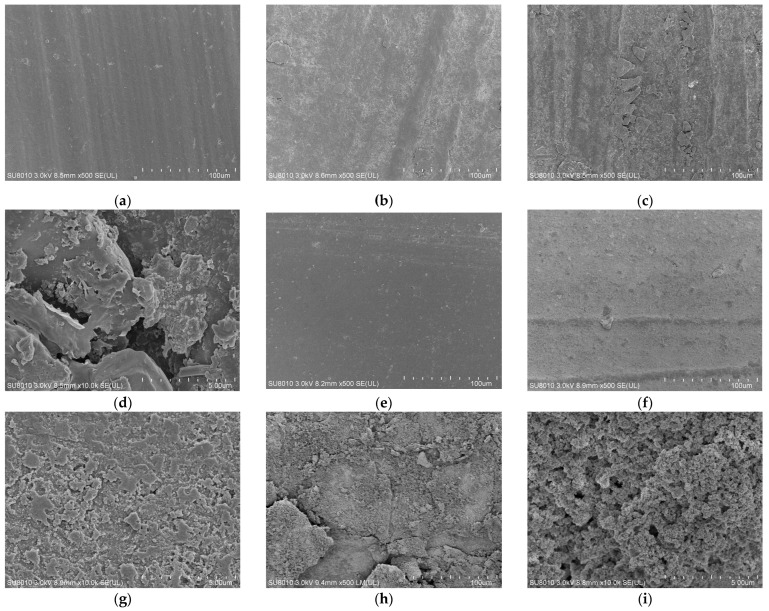
Surface morphology of FKM after hygrothermal–salt–compression aging. (**a**) FKM-A-NEW. (**b**) FKM-A-Hygrothermal-60 d. (**c**) FKM-A-Hygrothermal-Salt-60 d ×500. (**d**) NBR-A-Hygrothermal-Salt-60 d ×10,000. (**e**) FKM-B-NEW. (**f**) FKM-B-Hygrothermal-25%-60 d ×500. (**g**) FKM-B-Hygrothermal-Salt-20%-60 d ×500. (**h**) FKM-B-Hygrothermal-20%-60 d ×10,000. (**i**) FKM-B-Hygrothermal-Salt-20%-60 d ×10,000.

Figure 12 presents the micro-morphology of FKM dumbbell and seal specimens (at 20% compression) before and after hygrothermal and hygrothermal–salt aging. The FKM surfaces also became rougher and more porous after aging, though to a much lesser extent compared to NBR.

**Figure 13:**
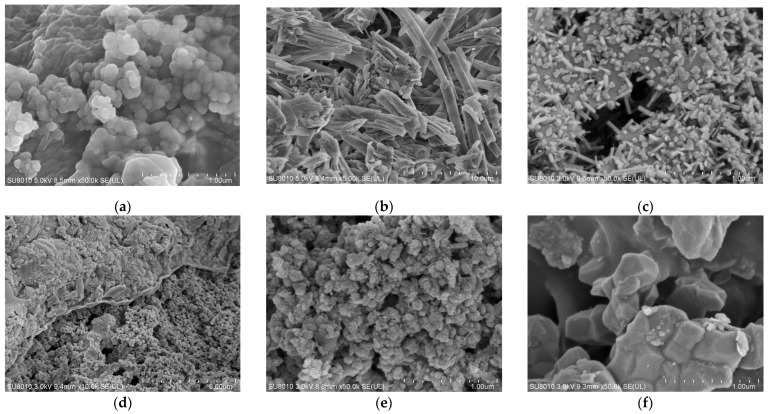
Micro morphology of partial characteristic regions. (**a**) NBR-A-Hygrothermal-Salt ×50,000. (**b**) NBR-B-Hygrothermal-Salt-20% ×5000. (**c**) FKM-B-Hygrothermal-20% ×50,000. (**d**) FKM-B-Hygrothermal-Salt-20% ×10,000. (**e**) FKM-B-Hygrothermal-Salt-20% ×50,000. (**f**) FKM-B-Hygrothermal-Salt-20%-inner ×50,000.

Detailed examination at higher magnification (×50,000) revealed distinctive morphological features, as shown in Figure 13. Spherical filler particles were extensively expelled after hygrothermal–salt aging, forming densely distributed clusters in certain areas. Spherical filler particles form during aging due to rubber matrix decomposition, humidity, and salt effects. On the outer surface of the seals, ribbon-like structures resulting from mechanical stress and harsh environmental conditions during aging were observed, along with small, aggregated rod-like and blocky particles. Clear boundaries between different structural domains and extensive blocky stacking were evident, indicating high surface inhomogeneity in both dumbbell and seal specimens after aging. The rubber matrix suffered apparent degradation, characterized by adsorption of external salts and rust, outward migration of internal fillers, and increased microporosity, collectively resulting in a severely damaged microscopic morphology.

### 3.4. Energy Dispersive X-Ray Spectroscopy (EDS) Analysis Under Different Aging Conditions

#### 3.4.1. EDS Analysis Under Thermo-Oxidative Aging

**Figure 14:**
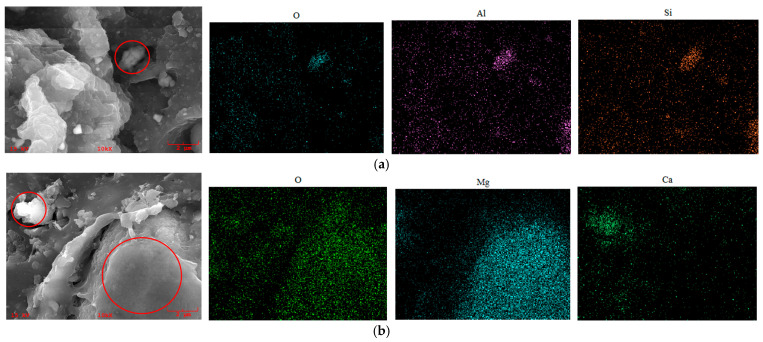
Elemental scanning distribution of aged sealing ring area after thermo-oxidative aging. (**a**) NBR. (**b**) FKM.

EDS analysis was performed on the internal cross-sections of both NBR and FKM seals before and after aging (80 days at 120 °C with 20% compression). The results are summarized in Table 2 and illustrated in Figure 14.

After aging, the relative atomic concentrations of oxygen (O), aluminum (Al), and silicon (Si) in NBR increased, while those of sulfur (S), calcium (Ca), and zinc (Zn) decreased. For FKM, the relative contents of oxygen (O) and magnesium (Mg) increased. The observed changes in NBR suggest the presence of additives, including calcium stearate, zinc oxide, and silane coupling agents. The significant increase in the O/C ratio in aged NBR indicates the progression of oxidation, resulting in deterioration of the internal structure and loss of elasticity. These findings are consistent with the conclusions drawn from the mechanical property analysis.

#### 3.4.2. EDS Analysis Under Hygrothermal–Salt–Compression Aging

**Figure 15:**
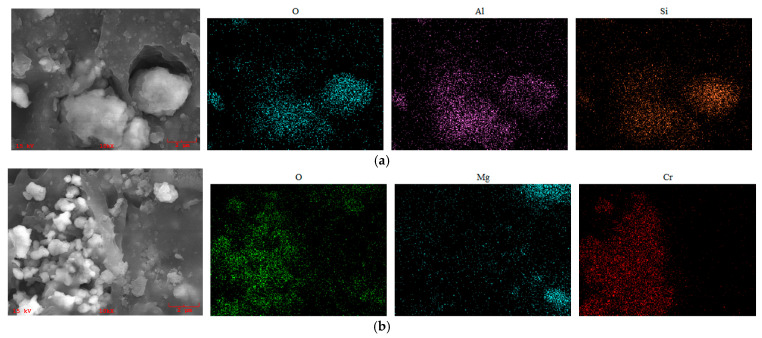
Elemental scanning distribution of aged sealing ring area after hygrothermal–salt–compression aging. (**a**) NBR. (**b**) FKM.

EDS area scanning was performed at ×10,000 magnification on the internal cross-sections of NBR and FKM seals after 60 days of hygrothermal–compression and hygrothermal–salt–compression aging. The elemental compositions of the scanned regions are designated as NBR-A (after hygrothermal–compression) and NBR-B (after hygrothermal–salt–compression) for nitrile rubber, and FKM-A (hygrothermal–compression) and FKM-B (hygrothermal–salt–compression) for fluoroelastomer, with the corresponding atomic percentages listed in Table 3. The elemental mapping results of the aged regions are presented in Figure 15.

For NBR, the aged samples showed increased concentrations of oxygen, aluminum, and silicon, along with a rise in the O/C ratio, indicating oxidation and degradation of the polymer matrix under hygrothermal aging. Numerous fillers detached from the matrix were observed, such as silane coupling agents and inorganic fillers containing Al and Si. In Figure 15a, the co-localization of O, Al, and Si signals corresponds to the detached white agglomerates visible in the micrograph. The loss of fillers can lead to their poor dispersion in the matrix, resulting in the deterioration of physico-mechanical properties such as tensile strength and abrasion resistance.

In the case of FKM, aging led to localized matrix damage, as reflected in the uneven distribution of fluorine. As shown in Figure 15b, local enrichments of magnesium, chromium, and oxygen were detected, presumed to be fillers such as magnesium oxide and chromate compounds. Their detachment from the matrix contributes to the degradation in FKM seal performance during aging.

### 3.5. Thermal Analysis Under Different Aging Conditions

#### 3.5.1. Thermal Analysis Under Thermo-Oxidative Aging

**Figure 16:**
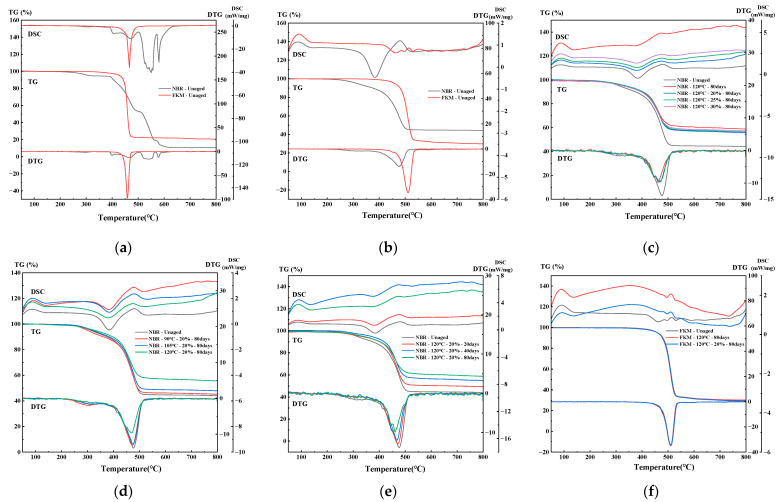
TGA of sealing ring under thermo-oxidative aging. (**a**) Oxygen atmosphere—New. (**b**) Nitrogen atmosphere—New. (**c**) NBR–Nitrogen atmosphere–different compression ratios. (**d**) NBR–Nitrogen atmosphere–different temperatures. (**e**) NBR–Nitrogen atmosphere–different aging time. (**f**) FKM—Nitrogen atmosphere.

Figure 16 shows the thermogravimetric (TG), derivative thermogravimetric (DTG), and differential scanning calorimetry (DSC) curves of NBR and FKM seals under different atmospheres, compression ratios, temperatures, and aging times.

As shown in Figure 16a, under an oxygen atmosphere, the unaged NBR exhibits multiple weight loss stages. Several weight loss and exothermic peaks appear between 380 and 600 °C, corresponding to the volatilization of small-molecule additives (e.g., plasticizers) and the severe thermo-oxidative degradation of the NBR backbone, which releases substantial heat. The curves stabilize beyond 650 °C, with the highest mass loss observed in oxygen and a residual mass of only 10.46%. In contrast, FKM remains highly stable below 400 °C, showing almost no mass loss due to the high bond energy and excellent chemical stability of C–F bonds. It exhibits a concentrated weight loss stage, with a sharp weight loss peak and exothermic peak between 441 and 483 °C, attributed to the thermo-oxidative cleavage of the fluorocarbon backbone.

Figure 16b–f present the results obtained under a nitrogen atmosphere for specimens aged under different conditions. NBR shows two main weight loss stages:

Stage I (200–350 °C): A weak weight loss peak and a relatively prominent exothermic peak are observed, corresponding to the decomposition and volatilization of small organic additives (e.g., plasticizers). After aging, the weight loss peak broadens and weakens, and the exothermic peak diminishes significantly, indicating additive loss and radical-induced crosslinking during aging. The reduction is more pronounced at higher temperatures and longer durations, with a substantial decrease in mass loss after aging at 120 °C for 80 days.

Stage II (400–530 °C): A sharp and prominent weight loss peak and a clear endothermic peak appear, corresponding to large-scale chain scission and matrix degradation. After aging, the peak broadens and weakens but remains notable. The peak decreases slightly at 90 °C and 105 °C but drops markedly at 120 °C, indicating chain scission and mass loss during prior aging.

Beyond 540 °C, the mass stabilizes. In nitrogen, complete pyrolysis does not occur, leaving residual carbon and inorganic fillers. Comparisons of total mass change indicate that high-temperature aging reduces total mass loss during TG testing, suggesting significant mass loss during prior aging. The influence of 120 °C is notably greater than that of 90 °C and 105 °C, resulting in substantial mass loss within just 20 days. Additionally, aging without compression leads to greater mass loss than with compression, consistent with macroscopic mass measurements.

FKM exhibits a sharp and concentrated weight loss peak between 450 and 550 °C, corresponding to large-scale degradation and backbone scission, accompanied by endothermic behavior. The total mass change before and after aging remains approximately 70%, indicating exceptional chemical stability throughout the aging process.

#### 3.5.2. Thermal Analysis Under Hygrothermal–Salt–Compression Aging

**Figure 17:**
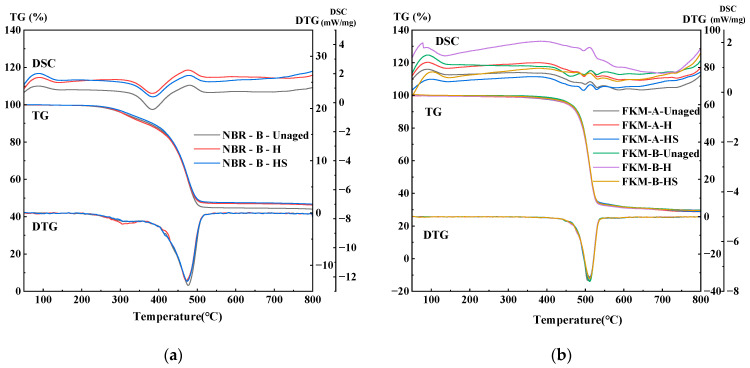
TGA of sealing ring under hygrothermal–salt–compression aging. (**a**) NBR—Nitrogen atmosphere. (**b**) FKM—Nitrogen atmosphere.

Figure 17 presents the thermogravimetric (TG) and heat flow curves of NBR and FKM seals before and after hygrothermal–salt–compression aging. The thermal decomposition of NBR in nitrogen occurs in two distinct stages:

Stage I (210–400 °C): A minor mass loss accompanied by a noticeable exothermic peak is observed, corresponding to the decomposition of additives (e.g., antioxidants and plasticizers). The unaged specimen exhibits greater mass loss at this stage, indicating that partial additive loss had already occurred during the prior aging process.

Stage II (410–535 °C): A sharp mass loss and a clear endothermic peak occur due to the large-scale pyrolysis of the polymer backbone. The similarity in this stage between aged and unaged specimens suggests that hygrothermal aging caused limited damage to the main chains.

Beyond 545 °C, the TG curves stabilize, leaving residues such as inorganic fillers and carbonized material. The slightly lower total mass change in aged specimens compared to unaged ones suggests that hygrothermal aging-induced mass loss was not significant.

In the case of FKM, neither the mass loss profiles nor the thermal peaks exhibit notable differences across aging conditions, and the total mass loss remains minimal, indicating exceptional thermal stability.

### 3.6. Fourier Transform Infrared (FTIR) Spectroscopy Analysis Under Different Aging Conditions

#### 3.6.1. FTIR Spectroscopy Analysis Under Thermo-Oxidative Aging

**Figure 18:**
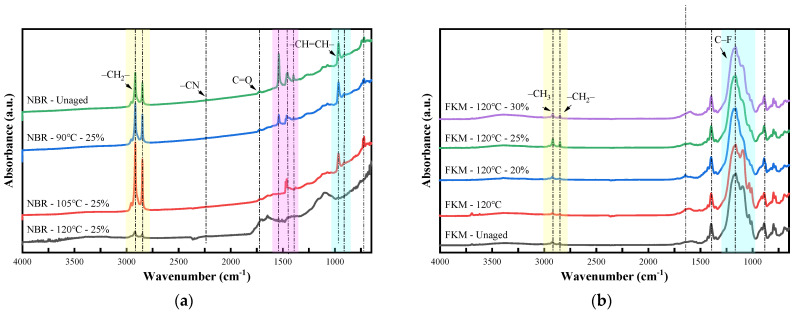
FTIR of sealing ring under thermo-oxidative aging. (**a**) NBR. (**b**) FKM.

Figure 18 shows the FTIR results of NBR and FKM under thermo-oxidative aging. For NBR, the double peaks at 719 cm^−1^ and 728 cm^−1^ increased after aging at 90 °C, significantly intensified after aging at 105 °C, and almost disappeared after aging at 120 °C. These peaks are characteristic signals of long-chain saturated alkyl groups. This indicates that, at the relatively low temperature of 90 °C, some small plasticizer and antioxidant molecules have volatilized, thereby exposing the long-chain structures, but the changes were minimal. When the temperature reached 105 °C, more low-molecular-weight plasticizers volatilized, and crosslinking reactions predominated without excessive chain scission, resulting in good retention of long chains and a significant intensification of the double peaks. At the high temperature of 120 °C, chain scission became the main reaction: high temperature accelerated the cleavage of NBR’s main chains, plasticizers were completely volatilized, and long-chain methylene structures were oxidized, leading to the almost complete disappearance of the double peaks. The –CH=CH– peaks at 966 cm^−1^ and 912 cm^−1^ slightly decreased after aging at 90 °C and 105 °C, but almost disappeared at 120 °C. This corresponds to crosslinking-dominated aging at lower temperatures (where more chain ends participated in reactions) and extensive chain scission and crosslinking at high temperatures.

A stretching vibration peak of –COOH at 1708 cm^−1^ appeared at 120 °C, corresponding to the formation of carboxylic acids during high-temperature reactions, which is a variation of carbonyl group (1720 cm^−1^) vibration. The peak at 1646 cm^−1^ increased significantly after aging at 120 °C, indicating the formation of –CONH_2_ under high-temperature conditions. The –CN peak at 2237 cm^−1^ showed no obvious decrease at 90 °C and 105 °C but almost disappeared at 120 °C, indicating that temperatures of 90 °C and 105 °C are insufficient to overcome the energy barrier for large-scale cyano group reactions. At 120 °C, extensive hydrolysis occurred, resulting in extensive cleavage of cyano-containing side chains. The sharp peaks of –CH_2_– at 2848 cm^−1^ and 2917 cm^−1^, as well as the –CH_3_ peak at 2956 cm^−1^, significantly intensified after aging at 90 °C, with even more prominent enhancement at 105 °C, but almost disappeared at 120 °C. This suggests that at 120 °C, the contents of –CH_3_ and –CH_2_– decreased sharply, accompanied by large-scale cleavage of alkyl main chains, destruction of the rubber structure, and loss of elasticity.

FKM exhibits excellent chemical structure stability. The broad peak in the range of 1089~1166 cm^−1^ in FKM is attributed to the stretching vibrations of various C–F bonds, the peak at 1398 cm^−1^ corresponds to the bending vibration of fluorine-containing methyl groups, and the peak at 887 cm^−1^ is the out-of-plane bending vibration of C–F bonds. These peaks showed minimal changes after aging under various temperatures and conditions. The structural stability of fluorine-containing functional groups during thermo-oxidative-compressive aging is attributed to the high bond energy of C–F bonds in FKM and its excellent resistance to thermo-oxidative aging, which prevents easy destruction of the main chain skeleton and fluorine-containing functional groups. The stretching vibration peak of carbon-carbon double bonds is located at 1600 cm^−1^, and the sharp peaks at 2838 cm^−1^ and 2917 cm^−1^ are the stretching vibration peaks of C–H bonds in –CH_2_– and –CH_3_ groups. These peaks showed only slight variation across different aging times, temperatures, and compression ratios. FKM’s dense molecular chains and stable key structures make it suitable for high-temperature environments, demonstrating its outstanding aging resistance.

#### 3.6.2. FTIR Spectroscopy Analysis Under Hygrothermal–Salt–Compression Aging

**Figure 19:**
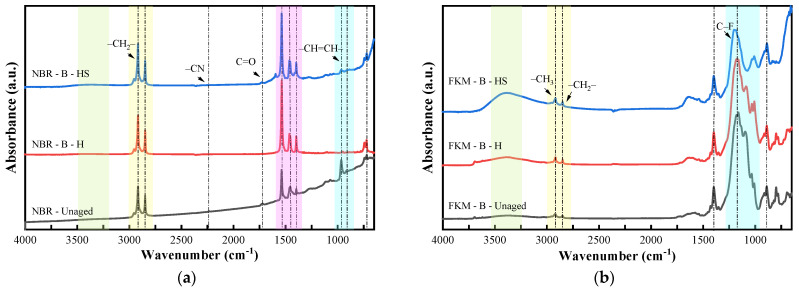
FTIR of sealing ring under hygrothermal–salt–compression aging. (**a**) NBR. (**b**) FKM.

Figure 19 shows the FTIR results of NBR and FKM under hygrothermal–salt–compression aging.

For NBR, the –CH_2_ peak in the range of 710–750 cm^−1^ increased after hygrothermal aging and hygrothermal–salt aging, indicating moderate crosslinking of molecular chains. The –CH=CH– peak at 966 cm^−1^ of the outer surface samples of the sealing rings was significantly weakened and almost disappeared after aging. Under the influence of humidity and temperature, the sealing rings underwent double-bond loss, oxidation, and crosslinking, which subsequently led to degradation of the butadiene main chain and more pronounced aging. The sharp peaks at 1398 cm^−1^, 1456 cm^−1^, 1463 cm^−1^, and 1538 cm^−1^ increased significantly after both hygrothermal aging and hygrothermal–salt aging, indicating additive migration to the surface, which aggravated aging. The reduction in the broad peak in the range of 1457–1463 cm^−1^ and the emergence of sharp peaks were attributed to the residual and migration of plasticizers, as well as to the regularization of the rubber matrix. The –CN peak at 2237 cm^−1^ on the outer surface of the sealing rings almost disappeared after hygrothermal aging and hygrothermal–salt aging. Extensive hydrolysis reactions occurred during aging, resulting in extensive cleavage of cyano-containing side chains, and humidity significantly accelerated cleavage of –CN bonds. The –CH_2_ peaks at 2848 cm^−1^ and 2917 cm^−1^, as well as the –CH_3_ peak at 2955 cm^−1^, increased significantly after aging, indicating molecular chain crosslinking. Humidity and salt accelerated oxidation and crosslinking, and the alkyl-containing small molecules generated by the slight hydrolysis of plasticizers under the action of humidity further enhanced this signal.

In the case of FKM, the broad peak in the range of 1087–1203 cm^−1^ includes characteristic peaks of various types of C–F bonds. This peak showed a pronounced decrease on the outer surface of the sealing rings after hygrothermal–salt–compression aging, indicating local degradation of the fluorocarbon skeleton. In contrast, it showed minimal changes under other conditions. The changes in the –CH_2_–, –CH_3_–, and –CH=CH– peaks were not significant. However, the broad -OH peak in the range of 3260–3520 cm^−1^ increased significantly after hygrothermal aging and even more prominently after hygrothermal–salt aging, whereas the -OH peak was almost undetectable in the unaged state. FKM itself contains a very low content of hydroxyl groups, indicating that moisture and temperature during aging led to moisture penetration and that oxidation and hydrolysis occurred on the rubber surface. Salt enhanced the moisture penetration rate, resulting in a substantial increase in hydroxyl group generation. Overall, most functional groups showed little change, demonstrating FKM’s excellent aging resistance.

## 4. Conclusions

This study systematically investigated the aging failure mechanisms of NBR and FKM transformer bushing seals under various single and multi-factor conditions. This is of great significance for the material selection, structural optimization, and maintenance strategies of transformer bushing sealing rings in complex environments subject to the combined effects of humidity, heat, and salt. Based on the experimental results and discussion, the following conclusions can be drawn:

(1) Material Performance Divergence: NBR is highly susceptible to thermo-oxidative and hygrothermal aging, exhibiting significant mass loss, a substantial increase in compression permanent set, severe surface cracking, and the detachment of fillers. FKM, in stark contrast, demonstrates superior resistance across all tested conditions, maintaining excellent mechanical integrity, thermal stability, and minimal mass change.

(2) Dominant Failure Mechanisms for NBR: After hygrothermal and hygrothermal–salt aging of NBR, the –CH_2_– peak increases, the –CH=CH– peak decreases, additive peaks intensify, the –CN peak almost disappears, and an –OH peak emerges—indicating molecular chain crosslinking and additive migration to the surface. At high temperatures, chain scission predominates, with extensive chain scission and crosslinking. These processes lead to hardening, loss of elasticity, and the formation of a brittle, fragile network structure, ultimately resulting in sealing failure.

(3) Synergistic Multi-Factor Effects: The coupling of multiple stressors does not represent a simple superposition of individual effects but creates a synergistic acceleration of degradation. For NBR, the combination of humidity, compression, and salt spray significantly intensified aging. The adsorption of salt particles increased osmotic pressure, facilitating deeper moisture ingress and exacerbating swelling and interfacial reactions, resulting in more severe surface erosion and an earlier onset of mass loss.

(4) Microstructural and Chemical Evidence: SEM and EDS analyses confirmed that multi-factor aging causes more profound microstructural damage, including increased porosity, filler migration, and element composition changes (e.g., increased O/C ratio in NBR). These micro-scale alterations directly correlate with the observed macro-scale deterioration in performance.

## Figures and Tables

**Table 1:** Mechanical Properties of Samples.

Aging Factor	Aging Time (d)	Sample	Mechanical Properties	
			Elongation at Break (%)	Tensile Strength (MPa)
Hygrothermal	0	NBR	235.40	17.37
		FKM	261.83	12.77
	20	NBR	205.95	18.74
		FKM	260.47	11.31
	40	NBR	208.38	18.04
		FKM	275.74	12.14
	60	NBR	205.56	18.12
		FKM	271.81	12.38
Hygrothermal–salt	60	NBR	185.12	17.03
		FKM	264.16	12.27

**Table 2:** Elemental analysis of NBR and FKM before and after thermo-oxidative aging.

Element	Content (%)			
	NBR-Unaged	NBR-Aging	FKM-Unaged	FKM-Aging
C	64.44	65.34	18.49	16.76
O	3.76	10.9	8.76	15.31
F	\	\	43.52	29.45
Mg	\	\	3.54	21.07
Al	3.83	4.79	4.32	2.77
Si	3.83	4.3	12.64	7.37
S	7.49	3.91	\	\
Ca	2.39	1.74	5.89	5.34
Zn	14.26	9.02	\	\
Cr	\	\	2.84	1.91

**Table 3:** Elemental analysis of NBR and FKM before and after hygrothermal–salt–compression aging.

Element	Content (%)					
	NBR-Unaged	NBR-A	NBR-B	FKM-Unaged	FKM-A	FKM-B
C	64.44	51.37	65.65	18.49	11.97	10.14
O	3.76	15.3	8.03	8.76	12.78	13.89
F	\	\	\	43.52	34.45	31.56
Mg	\	\	\	3.54	5.87	5.29
Al	3.83	9.28	4.85	4.32	3.21	2.24
Si	3.83	10.26	4.41	12.64	8.37	7.33
S	7.49	3.19	4.03	\	\	\
Ca	2.39	1.44	2.05	5.89	7.79	4.16
Zn	14.26	9.16	10.97	\	\	\
Cr	\	\	\	2.84	15.56	25.39

## Data Availability

The original contributions presented in the study are included in the article; further inquiries can be directed to the corresponding author.
